# Quantitative estimation and interpretation of non-technical loss severity in smart grids using RFE-optimized XGBoost regression

**DOI:** 10.1038/s41598-026-59116-3

**Published:** 2026-06-21

**Authors:** Vahid Parvaz, Jabbar Ganji

**Affiliations:** Department of Electrical Engineering, Mahshahr Branch, Islamic Azad University, Mahshahr, Iran

**Keywords:** Electricity Theft, Non-Technical Loss Quantification, High-Precision Regression, Recursive Feature Elimination (RFE), Prediction Interval (PI), Interpretability, Energy science and technology, Engineering, Mathematics and computing

## Abstract

Non-technical losses (NTL) remain a major source of economic risk for electricity distribution utilities. Although extensive research has focused on classification-based theft detection, such approaches typically provide binary decisions and offer limited support for quantifying the magnitude of consumption irregularities, which is essential for inspection prioritization and financial planning. This study proposes a regression-driven framework to estimate the severity of deviation from expected demand behavior using the Cumulative Abnormal Deviation (CAD) index. Instead of attempting theft verification, the method provides a continuous risk indicator derived from the difference between measured consumption and a baseline demand model. The framework combines Extreme Gradient Boosting with Recursive Feature Elimination to construct a compact predictor while preserving feature interpretability. Experiments conducted on a publicly available smart-meter dataset demonstrate that the optimized XGBoost-RFE model achieves high predictive consistency (R² = 0.9869, RMSE = 2.4062, MAE = 1.1285) and outperforms ensemble and deep learning benchmarks under identical data conditions. Residual-based prediction intervals show stable uncertainty behavior, with 93.83% of observations captured within the nominal 95% confidence bounds. Feature-attribution analysis indicates that load-related variables dominate deviation formation, supporting the operational plausibility of the model outputs. The results suggest that reliable estimation of deviation severity can be achieved under the evaluated experimental conditions without direct access to inspection outcomes. Nevertheless, the framework should be interpreted as a decision-support and prioritization tool, not as proof of theft. The proposed methodology provides a statistically grounded step toward quantitative, explainable, and practically applicable NTL risk assessment in modern distribution systems.

## Introduction

### Motivation and problem statement

Non-Technical Losses (NTL), arising from electricity theft, metering malfunctions, and administrative inconsistencies, continue to impose considerable economic pressure on distribution utilities. In some service territories, reported losses can exceed 20% of the delivered energy, affecting cost recovery, infrastructure planning, and system reliability^1,2^. The large-scale deployment of smart meters has enabled high-resolution monitoring of customer demand and has motivated the adoption of machine learning techniques for abnormal consumption analysis. These data-driven approaches have shown potential to improve the ability of utilities to detect suspicious behavior at scale.

However, in practical operations, a binary alarm alone is often insufficient. Field inspection, auditing logistics, and financial planning require prioritization, which depends not only on whether an anomaly exists, but also on its expected magnitude. Consequently, there is increasing interest in analytical tools capable of translating consumption irregularities into quantitative severity indicators that can assist decision-making under limited resources.

### Literature review

A substantial body of research has investigated electricity theft detection using supervised learning algorithms. Classical classifiers such as Support Vector Machines, Random Forests, and gradient boosting models have shown strong predictive performance on labeled datasets^3–6^. In parallel, deep learning architectures—including CNN–LSTM and adversarial frameworks—have been introduced to capture complex temporal dependencies and nonlinear patterns in load profiles^7–9^. These studies have advanced the state of automated detection and demonstrated that data-driven techniques can outperform manual rule-based screening.

Nevertheless, most contributions share a common characteristic: the task is formulated primarily as classification, producing discrete outcomes (e.g., normal vs. suspicious). While valuable for screening, such outputs do not directly inform the expected scale of deviation, nor do they readily support cost–benefit analyses of inspection campaigns. In addition, although interpretability methods are increasingly adopted, they are frequently used to rank variables rather than to relate predictions to operational decision variables. Formal treatment of predictive uncertainty is also less common, despite its importance for risk-aware planning.

Recent surveys emphasize the need to complement detection capability with models that can offer quantitative, transparent, and statistically reliable information for utility operators. The literature summarized in Table 1 illustrates strong maturity in anomaly identification but comparatively limited attention to deviation severity estimation.

**Table 1:** A review of studies conducted.

Ref.	Purpose	Innovation	Method	Results
^10^	To develop a machine learning-based system for detecting electricity theft among postpaid customers using traditional meters in Indonesia’s PLN network.	Introduced a multi-model sequential evaluation approach to enhance accuracy in forming Target Operation (TO) lists for field inspection.	Evaluated several ML models (Decision Tree, Naive Bayes, Random Forest, KNN, Logistic Regression, DNN); hybrid RF + KNN combined with layered filtering for enhanced detection.	Achieved accuracy = 0.89, precision = 0.83, recall = 0.98, F1 = 0.90, AUC = 0.89; improved efficiency and reduced human bias in TO selection.
^11^	To improve accuracy and robustness in electricity theft detection (ETD) using multi-temporal data features.	Proposed an attention-optimized, multi-temporal granularity convolutional ensemble model for robust ETD.	Two integrated modules: (1) SE-optimized Temporal CNN for temporal features, (2) CBAM-enhanced ResNet for spatio-temporal features; fusion and final classification via FC layer.	Demonstrated superior accuracy and generalization across datasets and imbalance ratios; validated on a Raspberry Pi for real-world deployment.
^12^	To reduce false detection rates in photovoltaic electricity theft classification.	Developed a novel 1D-CNN + BiLSTM hybrid model optimized by attention mechanisms (1DCNN-AMBL) and an objective optimization function for unbalanced data.	Deep learning architecture combining 1DCNN-AMBL with feature-level correlation analysis; trained on Ausgrid Power dataset.	Achieved lowest FPR = 0.61% and highest AUC = 98.46%; reduced false detection rate by an order of magnitude.
^13^	To detect electricity theft effectively in highly imbalanced datasets.	Employed ensemble learning with stacking and voting, combined with ADASYN for data balancing.	Stacking ensemble of Logistic Regression + Random Forest trained on ADASYN-balanced data (2014–2016).	Reached accuracy = 0.94, F1 = 0.94 for both theft/non-theft; ROC-AUC ≈ 0.94; stacking outperformed voting ensemble and baselines.
^14^	To enhance electricity theft detection accuracy using machine learning models with balanced datasets.	Utilized the ADASYN data augmentation technique to mitigate class imbalance and boost model reliability.	Tested multiple ML models (Naive Bayes, LR, SVM, AdaBoost, Decision Tree, Random Forest) on public datasets.	Random Forest achieved best results: 95% accuracy, precision, recall, and F1 for both theft and non-theft classes.
^15^	To provide a comprehensive review of data-driven approaches for electricity theft and anomalous power consumption detection.	Offered a systematic classification of ML, DL, and hybrid models, highlighting datasets, challenges, and future research directions.	Systematic literature review and taxonomy of ETD methods, privacy-preserving strategies, and application areas.	Delivered an integrated framework summarizing trends, limitations, and guidance for future ETD research.
^16^	To develop an interpretable and accurate model for theft detection in smart cities using balanced data.	Proposed the IGANN-ETD-SCBD-IGM model—an interpretable generalized additive neural network optimized via the Secretary Bird Optimization Algorithm (SBOA).	Combined RN-SMOTE for data balancing, RLKF for preprocessing, MSGWT for feature extraction, and IGANN for classification.	Achieved 99% accuracy, 94.12% precision, and 91.92% recall; significantly outperformed existing benchmark models.
^17^	To enhance electricity theft detection accuracy using deep learning on real smart grid data.	Introduced a CNN + LSTM hybrid model with LoRAS data augmentation to handle incomplete and imbalanced datasets.	Deep learning pipeline extracting spatial and temporal patterns from consumption data; trained on State Grid of China dataset.	Achieved overall accuracy = 97%; outperformed previous studies; individual results: 98.75% accuracy, 95.45% recall, F1 ≈ 97.7%.

### Research gap

Despite the significant progress reported in the literature summarized in Table 1, several important challenges remain insufficiently addressed.

First, most existing studies formulate electricity theft analysis as a classification problem rather than a quantitative estimation problem.

For example, Refs. ^10,13,14,16^ and ^17^ primarily aim to distinguish theft from non-theft using binary or multi-class labels, reporting metrics such as accuracy, recall, and F1-score. While effective for screening suspicious customers, these approaches do not quantify the magnitude of the deviation or potential financial loss, limiting their usefulness for inspection prioritization and cost–benefit analysis.

Second, predictive uncertainty is rarely quantified in existing machine-learning-based theft detection frameworks.

Studies such as Refs. ^11,12^, and^17^ report strong predictive performance and robustness across datasets or imbalance ratios, but they provide deterministic outputs without confidence intervals or uncertainty bounds. In operational environments, however, uncertainty-aware predictions are important for risk-sensitive planning and resource allocation.

Third, although some recent works employ complex deep-learning or ensemble architecture, their operational interpretability remains limited.

For instance, Refs. ^11,13^, and^17^ achieve high detection accuracy using convolutional, residual, or ensemble structures, yet they provide limited explanation regarding how individual consumption attributes influence model outputs. This restricts transparency and may reduce trust in real-world auditing applications.

Addressing these limitations does not replace existing detection models; rather, it extends them toward quantitative, transparent, and statistically reliable decision support.

### Objective and contributions

This work presents an application-oriented regression framework that estimates the degree of deviation from expected consumption behavior through the Cumulative Abnormal Deviation (CAD) index. The objective is not to certify theft events, but to generate a continuous indicator that can assist utilities in ranking and managing investigation priorities.

The main practical contributions of this study can be summarized as follows:

1. Regression-based reformulation of deviation severity:

CAD is used to transform abnormality assessment into a continuous estimation problem, enabling magnitude-based comparison among customers.

2. Compact predictive modeling through feature selection:

An XGBoost Regressor combined with Recursive Feature Elimination is employed to reduce redundancy while maintaining predictive stability.

3. Uncertainty-aware estimation:

Residual-based prediction intervals are used to provide an empirical measure of reliability for the estimated deviations.

4. Operational interpretability:

SHAP-based feature attribution is utilized to reveal which consumption attributes most influence deviation estimates, supporting transparent communication with field engineers.

Together, these elements provide a practical step toward integrating quantitative analytics into NTL management procedures. Unlike classification-based ETD frameworks, the proposed formulation explicitly models deviation magnitude, thereby linking anomaly analytics to quantitative operational decision-making.

## Methodology

### Framework overview

The objective of this work is to quantify the severity of non-technical losses (NTL) by moving beyond binary identification toward continuous deviation estimation. Instead of predicting whether a customer is fraudulent, the proposed framework estimates how far the measured consumption departs from an expected non-theft reference.

To ensure methodological rigor and prevent optimistic bias, the analytical pipeline is organized into sequential stages:

1. chronological data partitioning.
2. baseline consumption modeling using only normal behavior in the training horizon.
3. computation of the Cumulative Abnormal Deviation (CAD).
4. feature engineering and dimensionality reduction.
5. regression modeling and benchmarking.
6. translation of deviations into energy-loss quantities.
7. uncertainty and interpretability analysis.

All transformations are derived exclusively from the training data and subsequently transferred to the test partition.

### Dataset description and preparation

Experiments were conducted on the publicly available Low Carbon London smart meter dataset provided through the UK Data Service^18^, which has been frequently adopted in electricity-theft and smart-grid studies^5^. The dataset contains hourly measurements from several end-use categories together with inspection-derived labels.

Data preparation included:

- removal of samples with missing or undefined labels.
- exclusion of inactive meters with constant-zero profiles.
- filtering of corrupted readings.

After preprocessing, 560,655 valid observations remained. Each observation corresponds to a customer–timestamp record, representing the measured consumption at a specific time step together with its associated engineered statistical, temporal, and behavioral descriptors. In other words, the analytical unit in this study is not an aggregated customer profile, but an individual time-indexed consumption instance. Key characteristics of the dataset are summarized in Table 2. For each timestamp, total measured demand $\:{C}_{i,t}^{measured}$ was computed as the sum of all electricity end-use components.

**Table 2:** Dataset specifications.

Feature	Descriptions and Values	Ref.
Time period	Full year usage data (2014)	^5^
Sampling frequency	• Daily usage data (365 points ) • Monthly usage data (12 points)	
Number of subscribers	About 4,240 active subscribers (final number after filtering)	
Data volume	Includes 365$\:\times\:$4240 usage data for daily profiles and 12$\:\times\:$4240 for monthly profiles.	
Target	Multi-class classification in two protocols: • 6-class protocol (P6): includes 6 classes (Normal, Theft_2_, Theft_3_, Theft_4_, Theft_6_, Theft_7_). • 7-class protocol (P7): includes 7 classes (adding Theft_5_ class).	
Basic features	Time series consumption data and domain-specific attributes (such as Electricity: Facility, Fans: Electricity, etc.)	

### Chronological partitioning and leakage control

Because consumption evolves over time, random splitting can expose the model to future patterns during training. To avoid temporal leakage, records were ordered according to their occurrence index. The earliest 70% of samples were used for model development, while the most recent 30% formed the hold-out test set. No information from the evaluation horizon influenced baseline estimation, scaling, feature selection, or model training.

### Baseline consumption modeling

Quantitative deviation analysis requires an estimate of expected demand under regular behavior. A Random Forest regressor was trained only on customers labeled as normal within the training partition. This prevents theft-related distortions from entering the reference model while enabling it to capture habitual dynamics such as seasonality, weekly cycles, and customer-class characteristics. The output of this model defines the baseline consumption $\:{C}_{i,t}^{baseline}$.

This temporal-ordering principle was consistently maintained not only for the final train/test split but also during internal validation procedures such as feature selection and hyperparameter assessment.

However, this strategy may introduce sample-selection bias if customers labeled as theft differ systematically from normal customers in terms of load scale, behavioral patterns, or seasonal response. In such cases, the baseline model may be partially misspecified for those customers, potentially amplifying the resulting CAD estimates. This trade-off between contamination avoidance and representativeness should be considered when interpreting the severity scores.

To assess the sensitivity of the baseline model to sample-selection assumptions, an auxiliary experiment was conducted in which candidate normal samples were identified using an unsupervised Isolation Forest rather than inspection-derived labels. The resulting CAD estimates showed poor agreement with the label-based baseline (negative R² and high MAE), indicating that naive unsupervised filtering may fail to capture representative normal behavior. These findings suggest that while the label-based approach may introduce selection bias, fully unsupervised alternatives are currently less stable in this setting.

### Definition of the CAD target

Severity is measured by the relative deviation between metered and baseline demand (Eq. (1)):

1 $$\:{CAD}_{i,t}=\frac{{C}_{i,t}^{actual}-{C}_{i,t}^{baseline}}{{C}_{i,t}^{baseline}}\times\:100$$

Negative CAD values are consistent with under-registration potentially associated with theft.

This formulation converts anomaly detection into a continuous regression problem and enables ranking by magnitude.

### Feature scaling

To maintain a consistent preprocessing pipeline across all evaluated models, numerical inputs were standardized according to Eq. (2). Although tree-based models such as Random Forest and XGBoost are relatively insensitive to feature scaling, this step was particularly important for scale-sensitive methods including Support Vector Regression (SVR), PCA-based modeling, and the CNN–LSTM benchmark.

2 $$\:{x}^{{\prime\:}}=\frac{x-{\mu\:}_{train}}{{\sigma\:}_{train}}$$

where $\:{\mu\:}_{train}$ and $\:{\sigma\:}_{train}$ are computed solely from training data.

### Feature engineering

Two descriptor groups were constructed (Eqs. (3)-(10)):

1. **Statistical Descriptors**

These summarize the distribution and variability of the consumption profile over the available observation horizon:

- **Mean consumption**

3 $$\:{\upmu\:}=\frac{1}{\mathrm{T}}\sum\:_{t=1}^{T}{C}_{t}$$

- **Standard deviation**

4 $$\:{\upsigma\:}=\sqrt{\frac{1}{\mathrm{T}}\sum\:_{t=1}^{T}{({C}_{t}-{\upmu\:})}^{2}}$$

- **Maximum consumption**

5 $$\:{C}_{max}=\mathrm{m}\mathrm{a}\mathrm{x}\left({C}_{t}\right)$$

- **Minimum consumption**

6 $$\:{C}_{min}=\mathrm{m}\mathrm{i}\mathrm{n}\left({C}_{t}\right)$$

- **Skewness** (asymmetry of consumption distribution)
- **Kurtosis** (tail heaviness / peakness)
- **Range**

7 $$\:{C}_{max}-{C}_{min}$$

2. **Behavioral and temporal descriptors**

These capture regularity, seasonality, and short-term fluctuations:

- **Weekday/weekend ratio**

8 $$\:\frac{Average\:Weekday\:Load}{Average\:Weekend\:Load}$$

- **Peak-to-average ratio**

9 $$\:\frac{{C}_{max}}{\mu\:}$$

- **Hourly variation index**

10 $$\:\frac{1}{T-1}\sum\:_{t=2}^{T}\left|{C}_{t}-{C}_{t-1}\right|$$

- **Daily variation index**
- **Month index**
- **Day-of-week index**
- **Hour-of-day index**
- **Rolling baseline consumption estimate**

These descriptors were computed for each customer–timestamp observation using local historical consumption windows and contextual calendar information.

Recursive Feature Elimination (RFE) was then applied to this full feature pool. The final optimized model retained 10 variables, including the most informative load-related and temporal descriptors.

These capture regularity and change dynamics, including weekday–weekend ratios and inter-period variation indices.

The aim is to represent consumer behavior while keeping computational requirements compatible with large-scale deployment.

These engineered descriptors are computed for each customer–timestamp observation using the available local consumption history and contextual temporal information.

### Recursive feature elimination

Although ensemble trees are resilient to high dimensionality, redundant inputs may reduce stability and interpretability. Recursive Feature Elimination (RFE) was therefore applied within the training set using gradient boosting as the estimator. After each removal step, performance was evaluated using a blocked time-series cross-validation scheme based on forward-chaining validation. In each fold, the training subset strictly precedes the validation subset in time, thereby preserving temporal ordering and preventing information leakage. The optimal subset corresponded to the minimum average validation RMSE across folds. The optimal subset corresponded to the minimum validation error, resulting in 10 retained variables. The test data had no influence on this process.

### Regression models

Three regression paradigms were evaluated to verify robustness.

- **Proposed compact boosting model**

An XGBoost Regressor (Extreme Gradient Boosting Regressor) trained on the RFE-selected features, emphasizing strong nonlinear modeling capability, regularization support, and high predictive accuracy with moderate computational cost.

- **Ensemble baseline**

A Voting Regressor combining linear, kernel, neighborhood, and boosting learners.

Weights were assigned according to validation performance.

- **Deep learning benchmark**

A hybrid CNN–LSTM network in which convolutional layers capture short-term fluctuations and recurrent units’ model sequential dependencies.

Implementation details and hyperparameter settings are summarized in Table 3.

**Table 3:** Technical configuration and computing environment used.

Feature	Details
Programming Language	Python (≥ 3.9)
ML Framework	Scikit−learn (≥ 1.3)
Essential libraries	• XGBoost (for Boosting) • SHAP (for interpretability) • Pandas and NumPy (for data management)
Computational Hardware	• High-frequency multi-core processors (such as Intel Core i7 or higher) • At least 32 GB RAM
Computational Accelerator	It is recommended to use GPU-enabled libraries (such as NVIDIA CUDA toolkit) to accelerate XGBoost training and SHAP heavy computations.

The CNN–LSTM model was configured with a Conv1D layer (32 filters, kernel size = 2), followed by a dropout layer (rate = 0.2), an LSTM layer with 64 units, and a dense layer with 32 neurons. The model was trained using the Adam optimizer with a mean squared error (MSE) loss function for 10 epochs and a batch size of 256.

Table 4 also shows the initial engineered feature set used before RFE.

**Table 4:** Initial engineered feature pool used before RFE.

Feature Group	Feature	Description
Statistical	Mean	Average consumption
Statistical	Std	Consumption variability
Statistical	Max	Peak load
Statistical	Min	Minimum load
Statistical	Skewness	Distribution asymmetry
Statistical	Kurtosis	Distribution peakness
Statistical	Range	Max-Min
Behavioral	Weekday/Weekend ratio	Behavioral regularity
Behavioral	Peak/Average ratio	Peak intensity
Behavioral	Hourly variation	Short-term fluctuation
Behavioral	Daily variation	Inter-day fluctuation
Temporal	Month	Seasonal indicator
Temporal	Weekday	Weekly indicator
Temporal	Hour	Intraday indicator
Baseline-related	Baseline estimate	Expected normal load

Additional tree-based baselines including Random Forest, LightGBM, and CatBoost were considered for comparative evaluation due to their strong performance on structured/tabular datasets.

### Training objective

All regressors were optimized using Mean Squared Error (MSE) as the primary loss function. MSE was selected because larger CAD estimation errors may correspond to high-severity non-technical losses and therefore should be penalized more strongly during training. Its quadratic formulation increases sensitivity to large deviations and encourages the model to better capture rare but operationally critical abnormal-consumption cases. Alternative loss functions such as MAE or Huber loss may improve robustness against outliers; however, in this study MSE provided stable convergence and strong empirical performance (Eq. (11)):

11 $$\:MSE=\frac{1}{N}\sum\:_{i=1}^{N}({y}_{i}-{\widehat{y}}_{i}{)}^{2}$$

which penalizes large deviations more heavily, aligning with the economic consequences of underestimation.

The strong MAE and RMSE values reported in Table 5 indicate that the MSE-based training objective achieved a favorable balance between average-case accuracy and sensitivity to severe deviations.

### From CAD to energy loss

Predicted deviation percentages are translated into recoverable energy. First, expected true demand is reconstructed (Eq. (12)):

12 $$\:{\widehat{C}}_{i,t}^{actual}={C}_{i,t}^{baseline}\times\:\left(1+CA{D}_{i,t}/100\right)$$

The estimated non-technical loss is then computed as (Eq. (13)):

13 $$\:{NTL}_{i,t}=\mathrm{max}(0\:,\:{\widehat{C}}_{i,t}^{actual}-{C}_{i,t}^{\mathrm{m}\mathrm{e}\mathrm{a}\mathrm{s}\mathrm{u}\mathrm{r}\mathrm{e}\mathrm{d}})$$

ensuring non-negative energy estimates.

### Uncertainty quantification

To support operational decision-making, prediction reliability must be assessed. Let $\:{\sigma\:}_{res}$ denote the standard deviation of residuals observed on validation data. Approximate 95% prediction intervals are obtained as (Eq. (14)):

14 $$\:{PI}_{{\upalpha\:}-1}={\widehat{Y}}_{i}\pm\:1.96\times\:{\sigma\:}_{\mathrm{r}\mathrm{e}\mathrm{s}\mathrm{i}\mathrm{d}\mathrm{u}\mathrm{a}}$$

The empirical coverage of these intervals is reported later.

### Performance metrics

Accuracy is evaluated using MAE, RMSE, normalized RMSE, correlation, and $\:{R}^{2}$. Because CAD values are heavily concentrated near zero, MAE is emphasized during evaluation as an interpretable measure of average deviation and ranking accuracy, whereas RMSE reflects sensitivity to larger errors. Therefore, MAE and RMSE are reported jointly to provide complementary perspectives. So absolute-error measures receive primary emphasis to better reflect ranking capability.

### Interpretability

Model behavior is examined through SHAP-based feature attribution to determine which descriptors most strongly influence predicted deviation levels. These explanations represent statistical contributions and should not be interpreted as embedded physical laws.

## Experimental results and analysis

This section reports on the empirical performance of the proposed quantitative NTL assessment framework. The analysis proceeds from validation of the CAD target behavior to regression accuracy, dimensionality reduction, uncertainty evaluation, interpretability, and comparison with prior studies.

### Behavior of the CAD target variable

After preprocessing and baseline estimation, the final dataset comprised 560,655 samples (see Table 2). The empirical distribution of CAD exhibits a strong concentration around zero, with a long negative tail corresponding to potential under-registration. Two important observations emerge:

- First, the global mean CAD over the entire population equals − 4.76%, indicating that, on average, measured demand tends to fall below the baseline expectation. Such a shift may originate from technical losses, calibration imperfections, or persistent unauthorized consumption.
- Second, when disaggregated by inspection labels, customers marked as theft present an average CAD of − 11.66%, while the normal class remains approximately centered around zero.

This clear separation supports the suitability of CAD as a continuous proxy for deviation severity and justifies the transition from classification to regression.

### Evaluation of baseline regression models

The second phase focuses on evaluating the efficiency of baseline regression models in predicting the CAD target variable. The quantitative results from this analysis are presented in Table 5 and contain noteworthy points regarding the comparative performance of different models.

In addition to conventional baselines such as Linear Regression and SVR, stronger tree-based tabular-learning models including Random Forest Regressor, LightGBM, and CatBoost were evaluated under the same chronological split and metric definitions. Random Forest achieved robust performance (R² = 0.9536), confirming the effectiveness of ensemble bagging methods for nonlinear consumption behavior. LightGBM and CatBoost provided highly competitive results, achieving R² values of 0.9828 and 0.9844, respectively. Nevertheless, the XGBoost Regressor still achieved the best overall predictive performance with R² = 0.9859 and RMSE = 2.4946, while maintaining the lowest bias and strongest consistency.

Initially, linear models (Linear Regression and Support Vector Regression (SVR)) demonstrated relatively poor performance, with the R^2^ value near zero, indicating the inability of these methods to model the complex non-linear relationships in energy consumption. In contrast, the XGBoost Regressor achieved the best predictive accuracy among the evaluated models, achieving R^2^ = 0.9859 and RMSE = 2.4946. This result highlights the suitability of gradient boosting for capturing nonlinear consumption patterns in the dataset in extracting hidden patterns. The Voting Regressor, as an ensemble structure, achieved R^2^ = 0.9328, which is acceptable. However, its performance was slightly lower than the standalone XGBoost model, suggesting that the aggregation process, in this specific case, led to a relative reduction in overall predictive power. Finally, the evaluation of the Mean Bias Error (MBE) for the XGBoost model showed a value of -0.0023, which is nearly zero. This characteristic signifies the absence of any systematic bias in the predictions, indicating the absence of systematic over- or under-estimation for sensitive financial and operational applications.

To provide a more comprehensive evaluation, an additional widely used ensemble model, namely Random Forest, was incorporated into the baseline comparison. This allows for a more balanced assessment across different regression paradigms, including linear, kernel-based, and ensemble methods.

**Table 5:** Comparison of the performance of the basic regression models on the test set.

Regression model	MBE %	NRMSE %	RMSE %	MAE %	*R*	*R* ^2^
Linear Reg. (LR)	-0.0337	12.9149	19.9723	9.6863	0.3146	0.0988
Support Vector Reg. (SVR)	-3.9388	13.0407	20.1668	6.2327	0.3513	0.0812
Random Forest (RF)	-0.1142	2.9421	4.5514	1.9543	0.9891	0.9536
LightGBM Regressor	-0.0215	1.7824	2.7561	1.2875	0.9925	0.9828
CatBoost Regressor	-0.0187	1.7019	2.6317	1.2418	0.9928	0.9844
XGBoost Reg. (XGBR)	-0.0023	1.6131	2.4946	1.1806	0.9930	0.9859
Voting Regressor (Ensemble)	-0.9806	3.5259	5.4527	2.0635	0.9923	0.9328

The results indicate that ensemble-based methods significantly outperform traditional regression models. In particular, Random Forest achieves strong performance due to its ability to capture nonlinear relationships, although it remains slightly inferior to XGBoost in terms of overall accuracy. This confirms the effectiveness of gradient boosting techniques for modeling complex consumption patterns.

Preliminary tests indicated negligible performance differences for XGBoost with and without scaling, confirming that the main benefit of standardization was methodological consistency across heterogeneous models.

### Impact of feature selection

Applying Recursive Feature Elimination leads to a compact subset of ten variables. As reported in Table 6, this reduction:

- Improves RMSE from 2.4946 to 2.4062,
- Increases R² to 0.9869,
- Iowers MAE to 1.1285.

The gain, although moderate, indicates improved generalization and reduced variance. In contrast, PCA, with 95% retained variance, deteriorates performance, likely because orthogonal projections weaken the semantic relationships embedded in original descriptors. The CNN–LSTM benchmark, despite its representational capacity, achieves substantially lower accuracy (R² = 0.8227). This suggests that once informative features are engineered, deep sequential modeling may provide limited additional benefits relative to its computational cost.

**Table 6:** Performance comparison of optimized and benchmark models.

Model	MAE %	RMSE %	*R* ^2^	Usage Analysis
XGBoost with RFE (10 features)	1.1285	2.4062	0.9869	Best Final Model
XGBoost with PCA (16 components)	1.14653	3.1630	0.9774	Decreased accuracy vs. RFE
CNN-LSTM Hybrid	3.6416	8.8593	0.8227	Time series modeling capability, but lower accuracy

### Ablation study

To isolate the contribution of individual modules, an ablation study was conducted and summarized in Table 7. First, replacing the supervised baseline model with an unsupervised Isolation-Forest-based filtering strategy caused severe degradation (negative R²), highlighting the importance of reliable baseline estimation. Second, using XGBoost on the full feature set already yielded strong performance. Applying PCA slightly reduced MAE but substantially increased RMSE and reduced R², suggesting that orthogonal compression weakens meaningful feature semantics. In contrast, RFE improved both RMSE and R², confirming the benefit of targeted feature elimination. Finally, the CNN–LSTM benchmark underperformed compared with the proposed boosting-based framework, indicating that engineered tabular descriptors are more effective than deep sequential modeling under the current dataset conditions.

**Table 7:** Ablation analysis of major framework components.

Configuration	MAE	RMSE	*R*²	Observation
Unsupervised baseline + XGB	18.698	22.01	-5.46	Baseline unreliable
XGB (all features)	1.1806	2.4946	0.9859	Strong baseline
XGB + PCA	1.1465	3.1630	0.9774	Information loss
XGB + RFE	1.1285	2.4062	0.9869	Best configuration
CNN–LSTM	3.6416	8.8593	0.8227	Lower tabular performance

### Visual diagnostics of regression quality

**Fig. 1:**
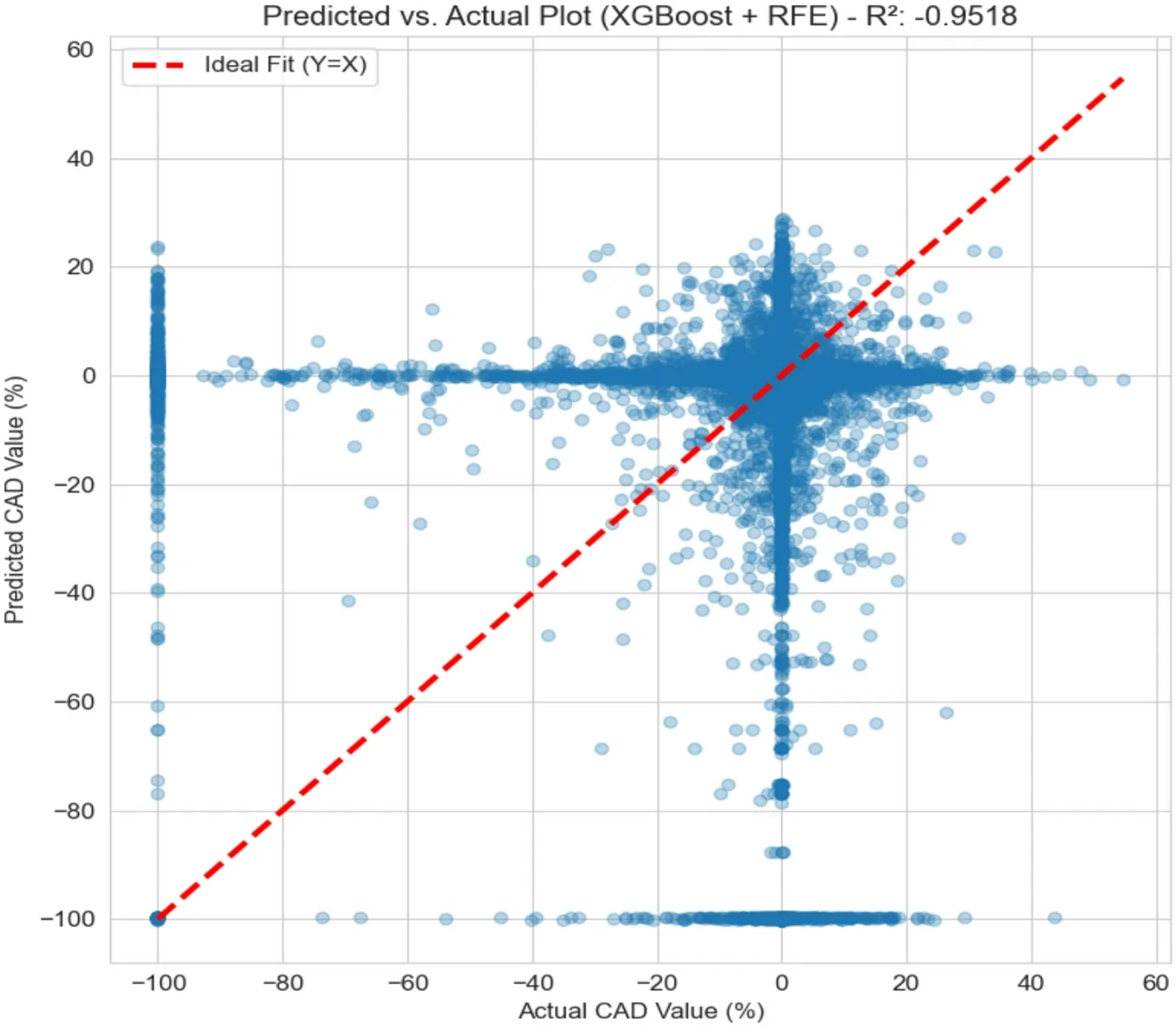
Scatter of actual vs. predicted CAD values (Actual CAD vs. Predicted CAD).

The scatter diagram in Fig. 1 shows a strong concentration of observations around the identity line, indicating close agreement between predicted and actual CAD values over most of the operating range. The clustering of points near the diagonal * y=x* suggests that the model reproduces the target variable with small deviations, which is consistent with the high coefficient of determination reported in Table 5. Furthermore, the dense accumulation of points at the center indicates high accuracy in predicting CADs close to zero (normal customers or small deviations), while the limited scattering of points at the extremities confirms the model’s stability in estimating high-severity CADs (large losses). This visual convergence strongly reinforces the reported high R^2^ value of 0.9869.

**Fig. 2:**
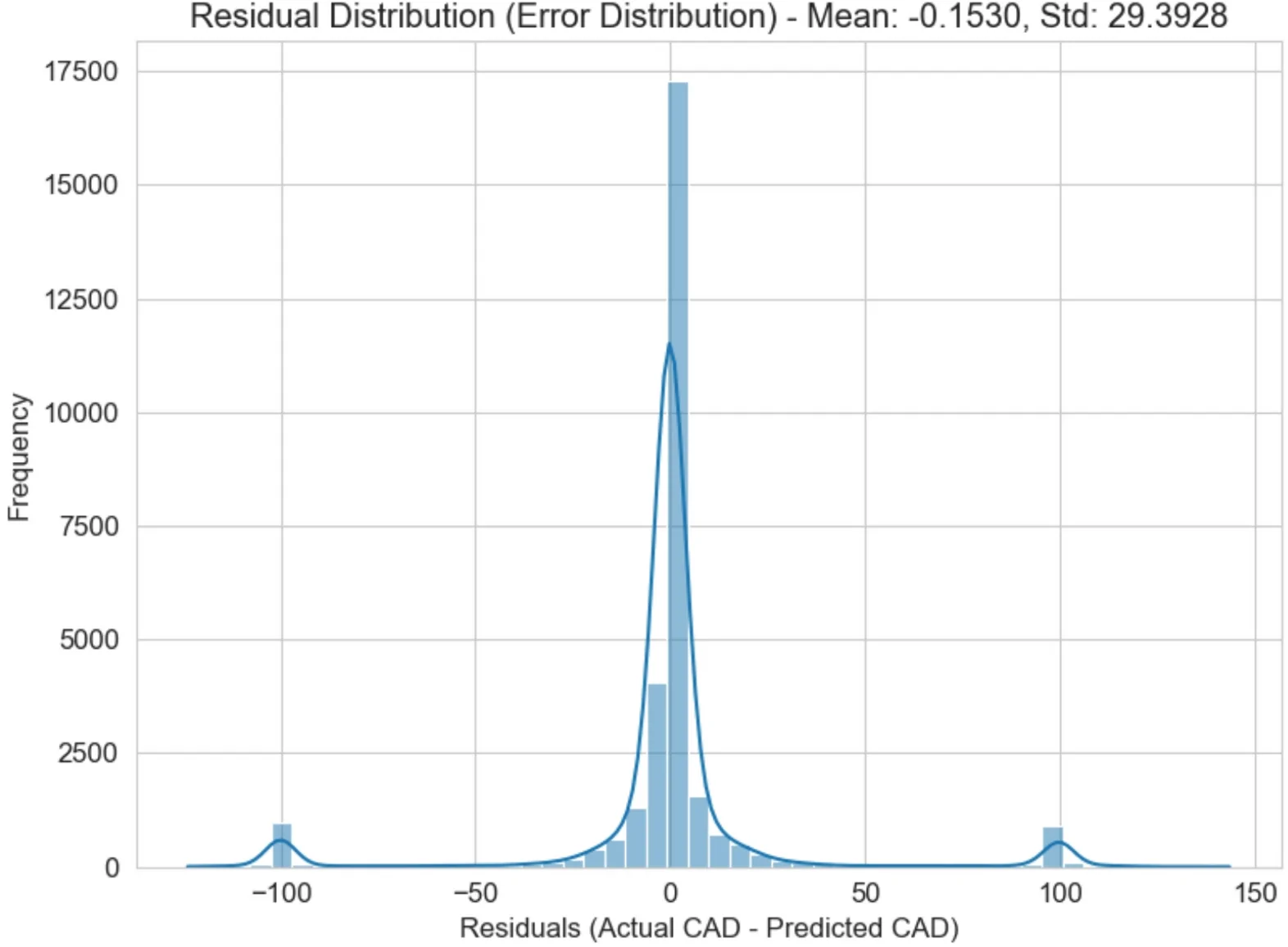
Regression Error Distribution.

Error histograms in Fig. 2 display an approximately symmetric bell-shaped pattern centered near zero, supporting the unbiased nature of the estimator and the validity of residual-based uncertainty analysis. This finding also indicates minimal systematic bias in the predictions (consistent with the near-zero MBE reported in Table 5). The alignment with the normal distribution significantly reinforces the validity of the resulting Prediction Interval (PI) analysis, in which 93.83% of the actual values fell within the 95% confidence bounds.

### Translation to energy-loss quantities

Regression outputs were converted into energy terms using the formulation introduced in Sect.  2. Across the evaluation horizon (≈ 30% of the dataset), the model identified 15,967 timestamps with positive estimated deviation, corresponding to a cumulative loss of:

- **Estimated NTL = 26**,**988.41 kWh**

Unlike binary detectors, the framework therefore provides both ranking capability and magnitude estimation, enabling prioritization of field inspections according to expected recovery.

### Prediction interval analysis

Uncertainty was assessed using residual-based prediction intervals (PI). Based on the final RMSE (2.4062), the 95% PI covered:

- **93.83% of actual CAD observations**

This level of coverage—while based on residual dispersion rather than full Bayesian uncertainty—suggests stable predictive behavior and provides a practical confidence measure for operational decision-making.

As an illustrative instance, a customer with an observed CAD of 0.01% received an interval of [-3.97%, 5.46%], within which the measurement was contained. This example is consistent with the aggregate coverage statistics and demonstrates how interval estimates may support audit decision workflows.

An additional experiment was conducted to evaluate the feasibility of label-free baseline modeling. An Isolation Forest-based approach was used to approximate normal samples without relying on labels. However, the resulting CAD estimates showed poor agreement with the supervised baseline (negative R² and high MAE), suggesting that unsupervised filtering alone is insufficient for reliable baseline construction in this context.

### Model interpretability and explainability analysis using SHAP

To improve the transparency and practical applicability of the proposed framework, the prediction mechanism of the final XGBoost-RFE model was interpreted using SHapley Additive exPlanations (SHAP). SHAP provides both global and local interpretability by quantifying the contribution of each input feature to the predicted Cumulative Abnormal Deviation (CAD) severity score.

**Fig. 3:**
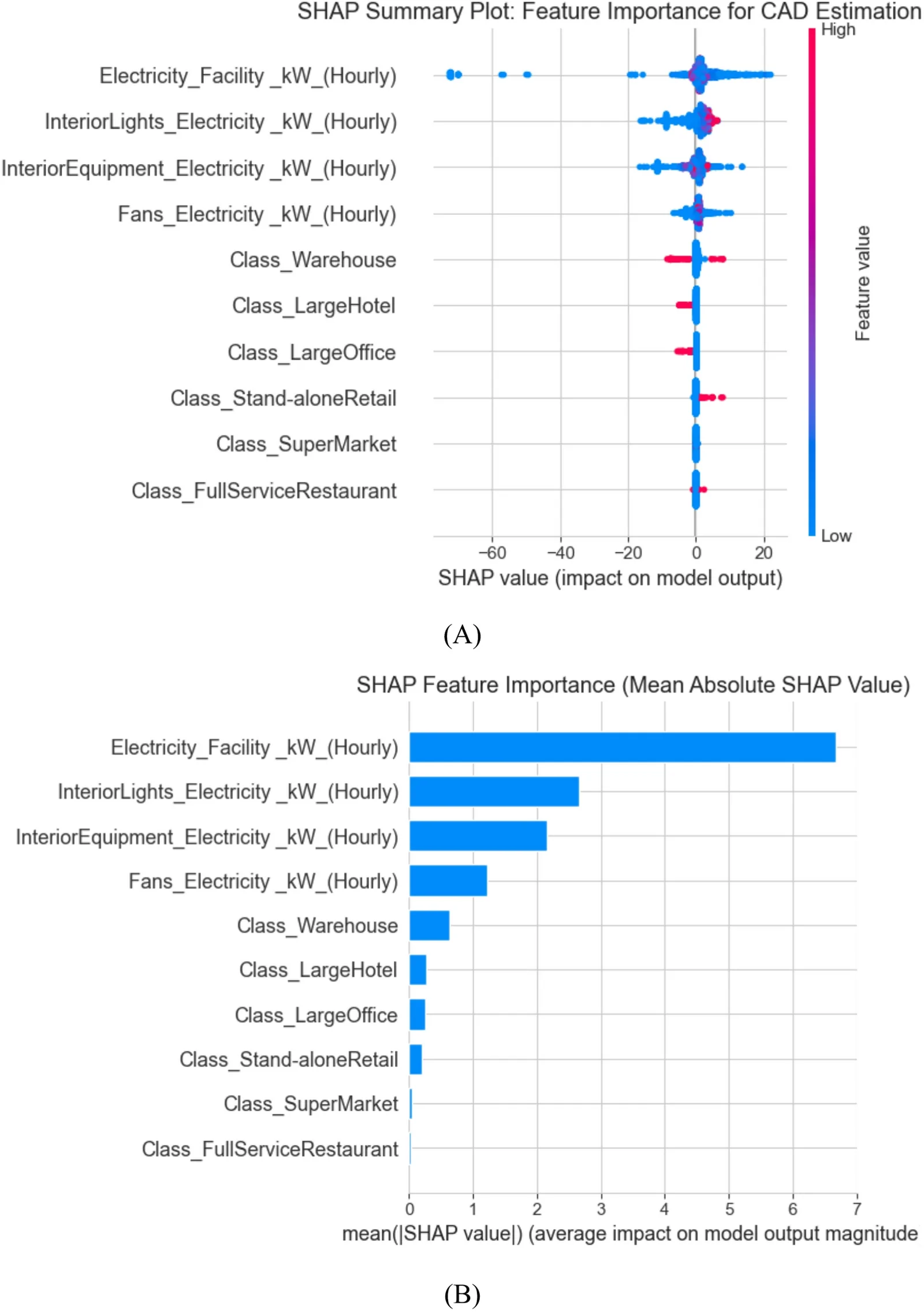
(**A**): Summary plot of SHAP values for the XGBoost − RFE model in CAD estimation, and (**B**): SHAP Dependence Plot.

SHAP analysis (Fig. 3(A)) indicates that temporal and load-related descriptors are the primary drivers of CAD predictions, where higher values positively correlate with deviation magnitude. In contrast, static features like building type show minimal influence. The mean absolute SHAP values (Fig. 3(B)) confirm that observed energy consumption is the most critical predictor of anomalies, while descriptive variables remain secondary.

**Fig. 4:**
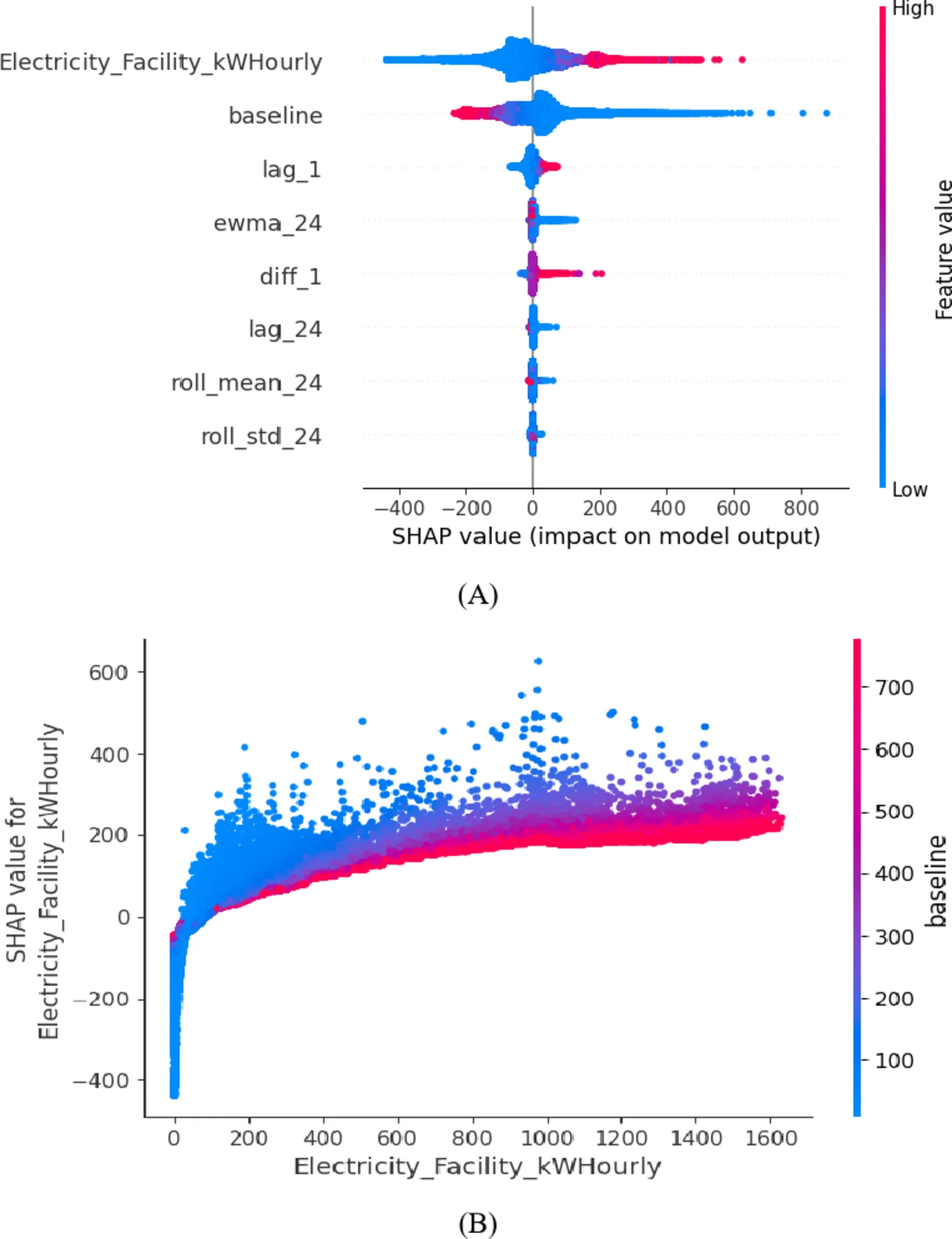

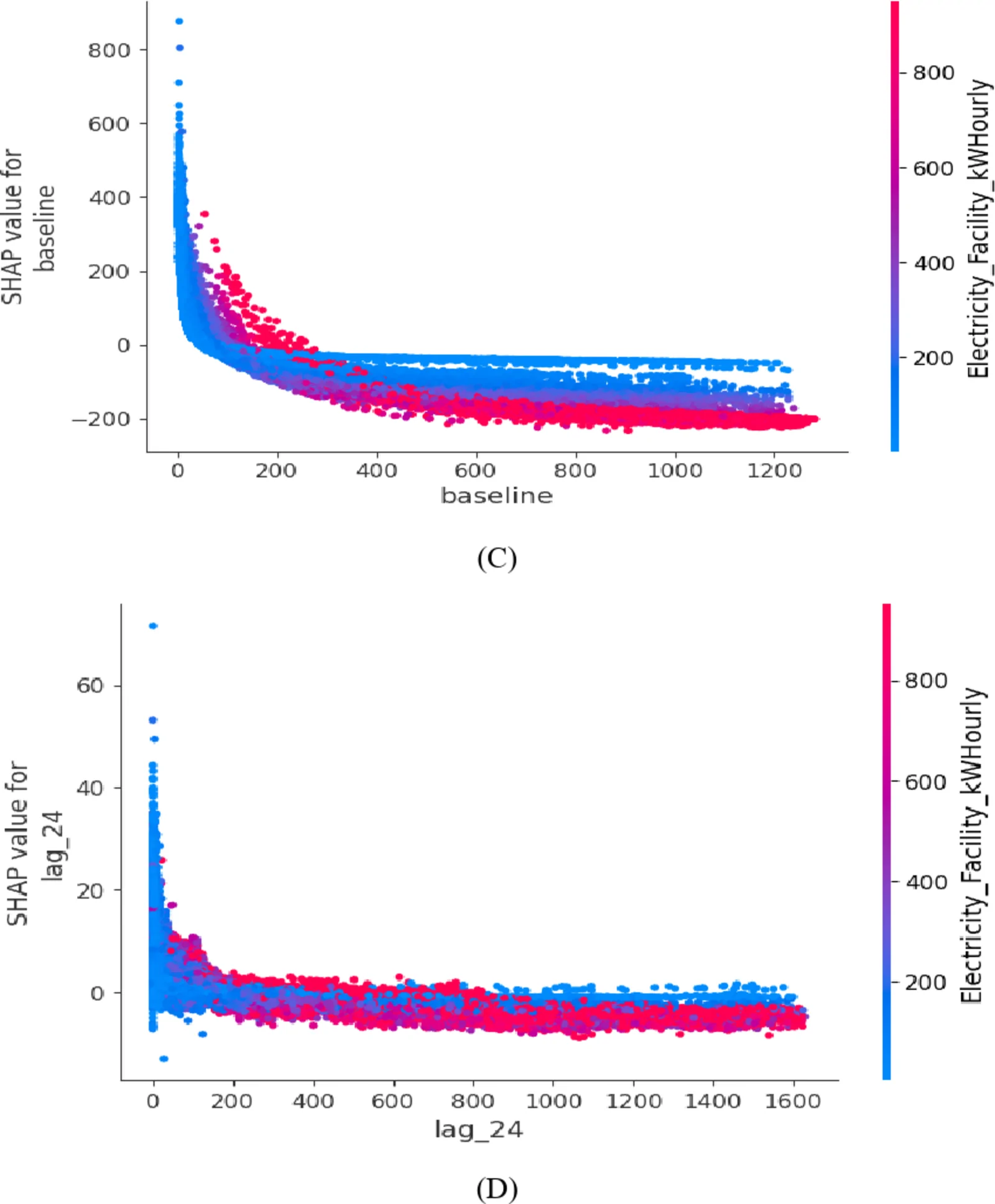
(**A**): SHAP summary plot showing global feature importance and directional influence on CAD prediction, (**B**): SHAP dependence plot for instantaneous electricity consumption, (**C**): SHAP dependence plot for baseline consumption, and (**D**): SHAP dependence plot for 24-hour lag consumption.

It can also be stated, Fig. 4(A) presents the SHAP summary plot, which illustrates the global importance ranking of the retained features and their directional influence on model output. Each point in the figure represents one observation, where the horizontal position indicates the SHAP value (i.e., contribution to the predicted CAD), and the color represents the feature value magnitude (red for high values and blue for low values). Features positioned toward the top of the figure have a larger overall contribution to the prediction process.

The results indicate that instantaneous electricity consumption is the most influential feature in predicting CAD severity. High values of electricity consumption generally push the prediction toward larger CAD values, suggesting that sudden or excessive deviations in real-time consumption are strongly associated with abnormal behavior severity. Similarly, lag-based and rolling statistical features, such as 24-hour lag consumption and rolling mean consumption, also show substantial influence, indicating that temporal consumption continuity and short-term behavioral trends play a critical role in abnormality assessment.

In contrast, baseline consumption exhibits a relatively stabilizing effect. Higher baseline values tend to reduce the predicted abnormal deviation severity, which is consistent with the definition of CAD as a deviation from expected normal consumption behavior. This confirms that customers with historically stable and high expected consumption are less likely to be assigned severe abnormality scores unless a significant deviation occurs.

To move beyond global feature ranking, SHAP dependence plots were further generated for the most influential variables, including instantaneous electricity consumption, baseline load, and 24-hour lag consumption (Fig. 4 (B)-(D)). These plots reveal nonlinear relationships and threshold-like effects between feature values and their contributions to CAD prediction.

For instance, the effect of instantaneous electricity consumption on CAD is not strictly linear. At lower consumption levels, its contribution remains relatively small; however, beyond a certain threshold, the SHAP value increases sharply, indicating that the model becomes significantly more sensitive to high-load anomalies. Similarly, the baseline feature demonstrates a nonlinear inverse relationship, where increasing baseline values reduce abnormality severity up to a saturation point. The lag_24 feature also shows non-monotonic behavior, suggesting that historical periodicity influences abnormality prediction differently across operating ranges.

Furthermore, the vertical dispersion of SHAP values for similar feature values indicates heterogeneity across customers and operating conditions. This suggests that the same feature value may contribute differently depending on contextual interactions with other temporal or behavioral features. Such heterogeneity highlights the importance of using an explainable nonlinear model rather than relying solely on simple threshold-based detection methods.

Overall, the SHAP analysis confirms that the proposed framework captures both instantaneous and historical consumption patterns, while also providing interpretable and actionable insights for utility operators. By identifying not only which features are important, but also how and under what conditions they affect CAD severity, the model can better support inspection prioritization and operational decision-making.

### Robustness and statistical stability analysis

To further evaluate the statistical reliability of the proposed model, the entire experimental pipeline was repeated five times with different random initializations. The resulting performance metrics are reported in terms of mean and standard deviation. The XGBoost–RFE model achieved MAE = 1.185 ± 0.015, RMSE = 2.506 ± 0.034, and R² = 0.9858 ± 0.0004. The very low variance across runs indicates high robustness and confirms that the observed performance is not dependent on a specific data split.

In addition, repeated experimental evaluations demonstrated that the proposed model maintains highly stable performance across multiple runs, further supporting its reliability for practical deployment.

Table 8 also shows statistical performance over multiple runs.

**Table 8:** Statistical performance over multiple runs (5 repetitions).

Model	MAE (%)	RMSE (%)	*R*²
XGBoost-RFE (Proposed)	1.185 ± 0.015	2.506 ± 0.034	0.9858 ± 0.0004

### Comparison with other studies

A structured comparison is provided in Table 9.

**Table 9:** Comparing the results of the proposed study with other previous studies.

Ref.	Issue / Scope	Main Method	Data / Time Horizon	Evaluation Metrics	Key Findings
^19^	Managing uncertainty and optimization in smart energy grids	Neutrosophic Logic–based optimization and anomaly detection model	Renewable generation, battery storage, and consumption data	Operating cost, net balance, detection accuracy	Reduced cost from USD 15–20 k to USD 8.5 k; improved scheduling, battery degradation prediction, and grid reliability
^20^	Detection and mitigation of DDoS attacks in networks	Machine learning (LSTM, SVM, Logistic Regression)	Real-time network traffic data	Accuracy, error rate	LSTM achieved 99.04% accuracy, outperforming SVM and Logistic Regression
^21^	Smart energy management and electricity theft prevention	IoT-based real-time monitoring + automated control	Real-time energy consumption and billing data	Billing accuracy, theft detection rate	Improved security and automation; enabled auto-disconnection and renewable integration
^22^	Smart theft detection via video surveillance	YOLOv8 + Generative AI + Streamlit interface	Real-time video feeds	Detection accuracy, response time	Fast and precise detection of unauthorized presence; real-time voice alerts for theft risk
^23^	Energy optimization through smart grid integration	Smart grid and AMI-based optimization	Energy consumption data across grid nodes	Energy efficiency, cost, reliability	Enabled load balancing, waste reduction, and improved decision support
^24^	Privacy-preserving smart grid management	Federated AIoT + Federated Graph Attention Network (FeGAN)	Nigeria’s smart grid data	Accuracy, F1-score, MCC, loss reduction	92% grid optimization accuracy, 89% theft detection, 15% energy loss reduction, MCC = 0.95
^25^	Role of AI in real-time and smart adaptive systems	AI (ML, DL, CV, Edge Computing)	Multi-sector real-time applications	Latency, scalability, accuracy	AI improves real-time decision-making in transport, healthcare, and smart cities
^26^	Electricity theft detection in regions with traditional metering and low-sampling-rate data	Data preprocessing + SMOTE + XGBoost	Real-world dataset from a northern Iranian province; 119,131 customers with 40–60-day interval meter readings	Accuracy, Precision	High accuracy (99.7%) and precision (98.2%) under sparse/noisy data
^27^	Detection of intermittent electricity theft attacks in AMI systems	LightGBM + Variational Bayesian Gaussian Mixture Model (VBGMM)	Smart meter electricity consumption data; interval-based temporal features	Detection Accuracy / comparative detection performance	Detects persistent/intermittent attacks; outperforms SOTA methods
^28^	Abnormal electricity consumption / theft detection with enhanced data quality	Label Cleaning via Clustering + LSH (LC-CLSH) + Negative Sample Generation (NSG-RWS) + ML-based detection	Real-world electricity consumption datasets	Detection Accuracy	Enhanced performance via label correction & sample generation
^29^	Capacity planning and charge control in hybrid energy storage systems	AI-driven forecasting and management	Renewable and hybrid storage system data	Energy cost, reliability, cycle life	Reduced cost and enhanced stability; real-time adaptive control
^30^	Scalable probabilistic load forecasting and clustering	UMAP + HDBSCAN + GMM + LSTM-Attention + XGBoost	High-frequency household energy & weather data	MAE, RMSE, CRPS, Pinball Loss	15% CRPS and 12% MAE reduction; weather integration + 9% MAE gain, attention + 8% boost
This Study	Quantification and interpretability of Non-Technical Losses (NTL) in smart grids	Optimized XGBoost-RFE Regression + SHAP interpretability analysis	Smart grid consumption & customer feature dataset	R² = 0.9869, RMSE = 2.4062, MAE = 1.1285, PI coverage = 93.83%	Direct NTL quantification (26,988.41 kWh), optimized feature set, high interpretability, superior performance over CNN-LSTM and SVR

A review of the studies analyzed in Table 9 indicates that over the last decade, research in the field of smart grids has gravitated toward integrating Artificial Intelligence (AI), Machine Learning (ML), and real-time data analysis. The core focus of these studies lies on two key axes:

1. Optimization and Stability of Energy Networks.
2. Detection or Prevention of Non-Technical Losses (NTL).

Studies such as ^19^ and ^23^, by leveraging intelligent models like Neutrosophic Logic and integrated frameworks, have focused on the economic optimization of the grid but lack a quantitative mechanism for NTL estimation. In contrast, research by ^21,22^, and^24^ has advanced toward automatic detection of theft or consumption anomaly, utilizing approaches like YOLOv8 and Federated AIoT, which have increased detection accuracy up to about 89\%. However, they remain at the binary classification level (theft/no-theft) and do not provide the capability for quantitative loss estimation. Furthermore, studies^31,32^, and^30^ have focused on increasing prediction accuracy (reducing RMSE) using hybrid models such as XGBoost, LSTM-Attention, and Active Learning. Nevertheless, these studies also lack the interpretability dimension and decision-support application concerning NTL.

In comparison, the present research, titled “Quantifying and Interpreting Non-Technical Losses in Smart Grids,” adopts a different methodological perspective. The proposed XGBoost–RFE framework not only identifies anomalies but also quantitatively estimates the actual volume of financial loss. The model’s numerical results (R^2^ =0.9869, RMSE = 2.4062, MAE = 1.1285) signify reports higher accuracy values under the present experimental setting compared to classification and hybrid models (such as CNN–LSTM). Furthermore, the uncertainty analysis, with a Prediction Interval coverage of 93.83%, confirms the model’s statistical stability and reliability. In addition, SHAP analysis is the key differentiating factor of this study, as, unlike opaque models, it transparently explains the influence of key consumption features (such as maximum power and consumption fluctuation) on loss severity.

Also, as shown in Table 9, most existing approaches focus on binary classification of theft behavior, whereas the proposed framework provides a continuous estimation of NTL severity along with uncertainty quantification and interpretability, offering a more informative decision-support tool for utility operators.

Overall, the comparative results show that the present research has extended existing studies by incorporating quantitative loss estimation alongside detection from mere detection toward explainable quantification of non-technical losses. The three characteristics of high accuracy, interpretability, and reliability of this framework make it an operational decision-support tool for network operators—a tool that has been absent in most previous studies and provides substantial operational capacity for energy auditing and policymaking. Likewise, the use of time-aware validation reduced the risk of optimistic bias and strengthened the robustness of the reported performance.

### Sensitivity analysis of model hyperparameters and feature set

To evaluate the robustness of the proposed framework, a systematic sensitivity analysis was conducted with respect to key XGBoost hyperparameters, including maximum tree depth, learning rate, subsampling ratio, and the number of estimators. In addition, the influence of feature selection was assessed by comparing the full feature set with RFECV-selected and reduced subsets.

The results indicate that the model achieves stable performance across a wide range of configurations. In particular, the R² score remains consistently within the range of approximately 0.72 to 0.76 for most configurations, with optimal performance observed for moderate tree depth (max_depth = 5–7) and intermediate learning rates (0.05–0.1). This suggests that the proposed framework is not highly sensitive to small variations in hyperparameters, which is desirable for practical deployment.

In contrast, aggressive feature reduction leads to a substantial degradation in performance ($\:R^2\:<\:0$), indicating that CAD prediction relies on a combination of temporal, lagged, and baseline-related features. This confirms the importance of engineered temporal descriptors in capturing consumption dynamics.

Overall, the sensitivity analysis confirms that the proposed XGBoost-RFE framework is both stable and robust, while highlighting the critical role of feature engineering in maintaining predictive accuracy.

### External validation on a second dataset

To assess the generalizability of the proposed framework, an additional validation experiment was conducted using the Low Carbon London smart meter dataset — provided through the UK Data Service — which reflects a different regional and residential consumption environment^18^.

Since this dataset does not include inspection-derived theft labels, the CAD target was approximated using a rolling-average baseline consumption model. The same regression framework was applied using temporal and baseline-derived features.

The model achieved MAE = 44.29, RMSE = 70.30, and R² = 0.125. Compared to the primary dataset, the reduced performance highlights the impact of domain shift, differences in consumer behavior, and the absence of supervision during baseline construction (Table 10).

Despite the lower predictive accuracy, this experiment provides an initial indication that the framework remains operational under heterogeneous conditions, while also revealing the need for transfer learning and domain adaptation techniques.

**Table 10:** External validation on a second dataset.

Dataset	MAE	RMSE	*R*²	Notes
Primary dataset	1.1285	2.4062	0.9869	Inspection-based supervised CAD

Preliminary external validation on the London Smart Meter dataset revealed performance degradation under distribution shift, emphasizing the importance of domain adaptation in future large-scale deployments.

## Conclusion and future work

### Conclusion

This study presented a quantitative and interpretable framework for assessing the severity of Non-Technical Losses (NTL) in smart grids. By reformulating electricity theft analysis from a binary detection task into a regression problem, the work introduced the Cumulative Abnormal Deviation (CAD) as a continuous indicator capable of expressing the magnitude of consumption irregularities rather than merely signaling their presence. The integration of gradient boosting with Recursive Feature Elimination (RFE) enabled the development of a compact yet highly expressive model capable of capturing nonlinear consumption deviations while preserving strong generalization performance. The empirical results on the evaluated datasets indicate that the resulting structure provides highly accurate CAD predictions accompanied by stable residual behavior, supporting its suitability for quantitative loss assessment in large-scale operational environments.

Beyond predictive accuracy, the framework allows direct estimation of customer-level and aggregated NTL volumes. This transition from anomaly flagging toward measurable loss quantification provides actionable information for inspection prioritization, auditing strategies, and resource allocation. Moreover, residual-based prediction interval analysis demonstrated statistically consistent behavior, offering an additional layer of reliability and helping decision-makers evaluate the confidence associated with each estimate. Interpretability was achieved through SHAP-based analysis, which showed that the dominant drivers of model predictions are physically meaningful consumption attributes, particularly facility-level demand and internal electrical subsystems. The alignment between model reasoning and realistic load behavior enhances trust, facilitates regulatory acceptance, and strengthens the applicability of machine learning outputs in practical utility workflows.

Experimental evidence also indicates that fully unsupervised baseline construction remains challenging and may lead to unstable CAD estimates. Therefore, developing robust semi-supervised or self-supervised baseline modeling strategies is an important direction for future work.

In addition, the current supervised baseline construction may be affected by sample-selection bias when applied to heterogeneous customer groups.

Also, ablation analysis further confirmed that the main gains arise from accurate baseline modeling and targeted feature selection.

Overall, the proposed methodology establishes a statistically grounded, explainable, and a framework suitable for preliminary practical evaluation for moving from detection-oriented paradigms toward quantitative NTL severity estimation. Despite the promising results, several limitations should be acknowledged. The proposed framework relies on the availability of high-resolution consumption data and a reasonably accurate baseline model. In environments with limited metering infrastructure or incomplete historical records, performance may degrade. Moreover, the residual-based prediction interval does not represent full probabilistic uncertainty, as it does not explicitly model heteroscedasticity. Addressing these aspects constitutes an important direction for future refinement.

### Future work

Future research can extend this work by:

- Developing adaptive baseline models resilient to seasonal and socio-economic variations.
- Conducting broader multi-region and multi-utility validation to assess transferability across different geographical and demographic contexts.
- Integrating Reinforcement Learning with XGBoost for dynamic feature selection and RFE optimization.
- Combining SHAP-based interpretability with financial risk metrics to estimate the Value at Risk (VaR) associated with NTL.
- Developing semi-supervised or domain-adaptive baseline models to reduce sample-selection bias and improve robustness across heterogeneous customer populations.
- Future work may investigate robust optimization objectives such as Huber or quantile loss for better handling of heavy-tailed CAD distributions.

## Data Availability

The Low Carbon London smart meter dataset used in this study is publicly available through the UK Data Service repository: Strbac, G., Tindemans, S., Woolf, M., Bilton, M., Carmichael, R. & Schofield, J. R. Low Carbon London Project: Data from the Dynamic Time-of-Use Electricity Pricing Trial, 2013 [data collection]. UK Data Service. SN: 7857, http://doi.org/10.5255/UKDA-SN-7857-2. Additionally, the processed data, including the feature-engineered daily and monthly consumption profiles used for regression and uncertainty analysis, have been deposited in the Mendeley Data repository and can be accessed at http://doi.org/10.17632/zsrzxdddmg.1.

## Abbreviations

R²: Coefficient of Determination
AMI: Advanced Metering Infrastructure
CAD: Cumulative Abnormal Deviation
CNN: Convolutional Neural Network
ETD: Electricity Theft Detection
GB: Gradient Boosting
LSTM: Long Short-Term Memory
MAE: Mean Absolute Error
MLP: Multi-Layer Perceptron
NTL: Non-Technical Losses
PI: Prediction Interval
PICP: Prediction Interval Coverage Probability
PINAW: Prediction Interval Normalized Average Width
RFE: Recursive Feature Elimination
RMSE: Root Mean Square Error
SHAP: Shapley Additive Explanations
XGBoost: Extreme Gradient Boosting

## Symbol

$\:{C}_{i,t}^{baseline}$: Measured electricity consumption at time * t*
$\:{\widehat{C}}_{i,t}^{actual}$: Baseline (predicted) normal consumption at time * t*
$\:{y}_{i}$: Actual CAD value for customer ( * i* )
$\:{\widehat{y}}_{i}$: Predicted CAD value for customer ( * i* )
* N*: Number of observations
* x*: Feature matrix
$\:{x}^{{\prime\:}}$: Standardization of inputs
$\:{\sigma\:}_{\mathrm{r}\mathrm{e}\mathrm{s}\mathrm{i}\mathrm{d}\mathrm{u}\mathrm{a}}$: Standard deviation of residuals
*α*: Significance level
$$\:{\mu\:}_{train}/{\sigma\:}_{train}$$: Educational data
* I*: Indicator function used in PICP calculation
$$\:{PI}_{{\upalpha\:}-1}$$: Forecast reliability

## Code availability

The custom Python codes and machine-learning workflows developed for data preprocessing, feature engineering, Recursive Feature Elimination (RFE), XGBoost regression modeling, uncertainty analysis, and SHAP-based interpretability are publicly available in the Mendeley Data repository at: https://doi.org/10.17632/zsrzxdddmg.2. The repository contains the implementation required to reproduce the main experimental results reported in this study.
